# *Terminalia bellirica* fruit extracts: in-vitro antibacterial activity against selected multidrug-resistant bacteria, radical scavenging activity and cytotoxicity study on BHK-21 cells

**DOI:** 10.1186/s12906-018-2382-7

**Published:** 2018-12-07

**Authors:** M. Priyanga Jayamal Dharmaratne, Amirthasingam Manoraj, Vasanthi Thevanesam, Asela Ekanayake, Nimal Savitri Kumar, Veranja Liyanapathirana, Eranga Abeyratne, B. M. Ratnayake Bandara

**Affiliations:** 1 Department of Chemistry, Faculty of Science, University of Peradeniya, Peradeniya, Sri Lanka; 2 Department of Microbiology, Faculty of Medicine, University of Peradeniya, Peradeniya, Sri Lanka; 3 National Institute of Fundamental Studies, Hantane, Kandy, Sri Lanka; 4 Animal Virus Laboratory, Veterinary Research institute, Polgolla, Sri Lanka

**Keywords:** *Terminalia bellirica*, Multidrug-resistant bacteria, Antibacterial, Antioxidant, Total phenolic content, BHK-21 cells

## Abstract

**Background:**

Identification of novel sources for developing new antibiotics is imperative with the emergence of antibiotic resistant bacteria. The fruits of *Terminalia bellirica* (Gaertn) Roxb., widely used in traditional medicine, were evaluated for antibacterial activity against multidrug-resistant (MDR) bacteria, antioxidant activity and cytotoxicity.

**Methods:**

Twelve solvent extracts of *T. bellirica* fruits were prepared by direct aqueous extraction and sequential extraction with dichloromethane, methanol and water using Soxhlet, bottle-shaker and ultrasound sonicator methods. Antibacterial activity of the extracts was tested against 16 strains MDR bacteria—methicillin-resistant *Staphylococcus aureus* (MRSA), extended spectrum β-lactamase (ESBL) producing *Escherichia coli* and MDR *Acinetobacter* spp., *Klebsiella pneumoniae* and *Pseudomonas aeruginosa*—and 4 control organisms, using the cut-well diffusion method. The minimum inhibitory concentration (MIC) was determined using an agar dilution method. The radical scavenging activity of six antibacterial extracts was screened against 2,2′-diphenyl-2-picrylhydrazyl (DPPH) and correlation was established between EC_50_ (50% effective concentration) values and the total phenolic content (TPC). Cytotoxicity was determined for the most potent antibacterial extract on baby hamster kidney (BHK-21) cells by Tryphan Blue exclusion method. Statistical analysis was carried out by one-way analysis of variance at significant level *p* < 0.05 using “SigmaPlot 10” and “R 3.2.0” software.

**Results:**

All aqueous and methanol extracts displayed antibacterial activity (MIC 0.25–4 mg/mL) against all strains of MRSA, MDR *Acinetobacter* spp. and MDR *P. aeruginosa*. The sequential aqueous extracts (MIC, 4 mg/mL) inhibited ESBL producing-*E. coli*. None of the extracts exhibited activity against MDR *K. pneumoniae* (MIC > 5 mg/mL). The sequential methanol extract (Soxhlet) recorded high antibacterial activity and the highest DPPH radical scavenging activity (EC_50_, 6.99 ± 0.15 ppm) and TPC content (188.71 ± 2.12 GAE mg/g).

The IC_50_ (50% inhibition concentration) values of the most potent antibacterial extract—the direct aqueous extract from reflux method—on BHK-21 cells were 2.62 ± 0.06 and 1.45 ± 0.08 mg/ml with 24 and 48 h exposure, respectively.

**Conclusions:**

Results indicate that *T. bellirica* fruit is a potential source for developing broad-spectrum antibacterial drugs against MDR bacteria, which are non-toxic to mammalian cells and impart health benefits by high antioxidant activity.

## Background

Emergence of MDR bacteria due to increased use and misuse of antibiotics poses a major health problem of global concern [1, 2]. Infection by MDR bacteria increases morbidity and mortality and requires increased expenditure to manage patients and implement infection control measures [3]. All current antibiotics available in clinical practice experience antibiotic resistance and none of them are effective against all MDR pathogens [4]. The World Health Organization (WHO) has prepared a priority list of antibiotic-resistant bacteria to guide research, discovery and development of new antibiotics and this list includes MDR *Staphylococcus aureus*, *Pseudomonas aeruginosa*, *Acinetobacter baumannii*, *Klebsiella pneumoniae* and *Escherichia coli* [5].

*S. aureus*, a Gram-positive bacterium, is a common cause of bloodstream infection, skin and soft-tissue infection and post influenza pneumonia. The efficacy of antibiotics in the control of this bacterium is fading because of the rapid emergence of MDR strains [1]. *P. aeruginosa* and *A. baumannii* are Gram-negative bacteria and opportunistic pathogens that can cause a range of infections including ventilator-assisted pneumonia, bacteraemia, endocarditis, meningitis, skin and soft tissue and urinary infections. *K. pneumoniae*, a Gram-negative bacterium present in the normal flora of the intestines, can cause bloodstream, urinary and respiratory infections and has emerged as a cause of MDR infections worldwide [1]. Several MDR mechanisms are known for Gram-negative bacteria such as *P. aeruginosa*, *A. baumannii*, *K. pneumoniae* and *E. coli* [4, 6].

The search for new antimicrobials and cost-effective strategies to combat antibiotic resistance is of increasing urgency. Medicinal plants, the major source for drugs in traditional medicine, have many therapeutic properties and, being accessible to poor communities of the world, provide an economically effective means of treatment for many serious diseases [7]. WHO estimates that 40–80% of people in Asia, Africa, China and Latin America depend on traditional medicine for their primary health needs, while traditional medicine, also referred to as complementary and alternative medicine, is becoming increasingly popular in many developed countries [8]. Medicinal plants can serve as a potential source of new antimicrobials to mitigate the problem of antibiotic resistance [9].

The dried ripe fruit of *Terminalia bellirica* Roxb. (Combretaceae) has traditionally been used in the treatment of diarrhoea, cough, hoarseness of voice, eye diseases and scorpion-sting and as a hair tonic. A decoction of the fruit is used for treating cough and pulp of the fruit is useful in treating dysenteric-diarrhoea, dropsy, piles and leprosy [10]. Fruit and fruit extracts of *T. bellirica* have shown a range of pharmacological activities, including antidiabetic, analgesic, antiulcer, antifungal, antibacterial and anti-hypertensive activity through in-vitro and in-vivo studies [11–16]. *T. bellirica* is one of the three ingredients of the well-known drug Triphala, used routinely in Ayurvedic medicine to treat a wide variety of diseases. *T. bellirica* is also widely used in Unani, Siddha and Chinese systems of traditional medicine [17].

The aqueous and methanol extracts of *T. bellirica* fruits have shown antibacterial activity against *S. aureus* (ATCC 9144), *Salmonella enterica* serovar Typhi (NCTC 8393), *Salmonella typhimurium* (ATCC 23564), *Pseudomonas aeruginosa* (ATCC 25619), *Yersinia enterocolitica* (ATCC 9610) and *Escherichia coli* obtained from urinary tract infections [14]. However, the effect of *T. bellirica* extracts on MDR bacteria has not been investigated.

Oxidative stress—that results when oxidation of cellular components by free radicals and reactive oxygen species exceeds antioxidant reactions—is implicated in several pathologies which include atherosclerosis, cancer, diabetes, obesity and neurogenerative diseases such as Alzheimer’s disease and Parkinson’s disease [18]. Extracts of *T. bellirica* fruits are likely to offer protection against oxidative stress, having potent antioxidant properties [11, 19–21]. A 70% methanol extract has effectively reduced free radicals and reactive oxygen species such as 1,1-diphenyl-2-picrylhydrazyl radical (DPPH), hydroxyl, superoxide, nitric oxide, peroxynitrite, singlet oxygen and hypochlorous acid in in vitro studies and increased the activity of antioxidant enzymes such as superoxide dismutase, catalase, glutathione S-transferase and glutathione reductase in mice [21]. The antioxidant activity of the fruit extracts correlates with their total phenolic content (TPC) indicating that phenolic compounds in the fruit contribute to the antioxidant activity [11, 19, 21]. Antioxidant properties of antibacterial extracts can confer additional health benefits.

Toxicity evaluation is an integral part of the assessment of plant products in developing therapeutic preparations. In vivo studies reveal that neither aqueous extract [22] nor an aqueous acetone extract [23] of *T. bellerica* fruits has been toxic to rats. In an in vitro study on mouse cell culture models, a hydroglycol extract of the fruit has been non-toxic to normal mouse fibroblast cells and mouse melanoma cells [24].

We hypothesized that extracts of *T. bellirica* fruits inhibit MDR bacterial pathogens and that the antibacterial extracts are non-toxic and have antioxidant properties. Our objectives were to assess 1) the antibacterial activity of 12 different *T. bellirica* fruit extracts—prepared by 3 direct and sequential extraction procedures using dichloromethane, methanol and water under ambient and hot conditions—against 16 selected MDR bacterial strains of *Acinetobacter* spp., *E. coli*, *K. pneumoniae*, *P. aeruginosa* and *S. aureus*, 2) the antioxidant activity of the antibacterial extracts using DPPH and its correlation with TPC and 3) the in vitro cytotoxicity of the most potent antibacterial extract on baby hamster kidney (BHK-21) cells.

## Methods

### Plant materials and microorganisms

*T. bellirica* dried fruit samples were procured from a traditional medicine store in Kandy, Sri Lanka and authenticated by Mrs. N.P.T. Gunawardena, a plant taxonomist at National Herbarium at Peradeniya Royal Botanical Garden, Sri Lanka. A voucher specimen (# 816) has been deposited in the National Herbarium at Peradeniya Royal Botanical Garden. The bacterial strains, *S. aureus* ATCC 25923 and NCTC 6571, *E. coli* ATCC 25922 and extended spectrum β-lactamase (ESBL)-producing *K. pneumoniae* ATCC 700603 and MDR bacteria were obtained from the archives of the Department of Microbiology, Faculty of Medicine, University of Peradeniya. The MDR bacterial strains have been isolated from clinical samples and saved in the archives of the Department of Microbiology as a part of routine practice. Since the isolates are used as a source of bacterial strains only, the isolates stay anonymous and the records are not associated with specific pathology or specific individuals. The bacterial strains were handled following the standard health/safety procedures. All bacterial strains were stored at − 81 °C (Thermo Scientific, USA). Disc diffusion antibacterial sensitivity testing was carried out on 35 bacterial isolates according to the Clinical and Laboratory Standard Institute method [25] with antibiotics representing different classes of antibiotics: penicillins (ampicillin), cephalosporins (cefuroxime, cefotaxime, ceftazidime, cefipime), carbapenems (imipenem, meropenem), quinolones (ciprofloxacin), monobactams (aztreonam) and aminoglycosides (amikacin, netilmicin, gentamicin). The following MDR bacterial strains were used for screening aqueous and organic extracts of *T. bellirica* fruit for antibacterial activity: 8 strains of MRSA with minimum inhibitory concentration (MIC) of oxacillin ≥128 mg/L and 2 strains each of ESBL-producing *E. coli*, MDR *Acinetobacter* spp.*,* MDR *K. pneumoniae* and MDR *P. aeruginosa*.

### Preparation of extracts

**Fig. 1:**
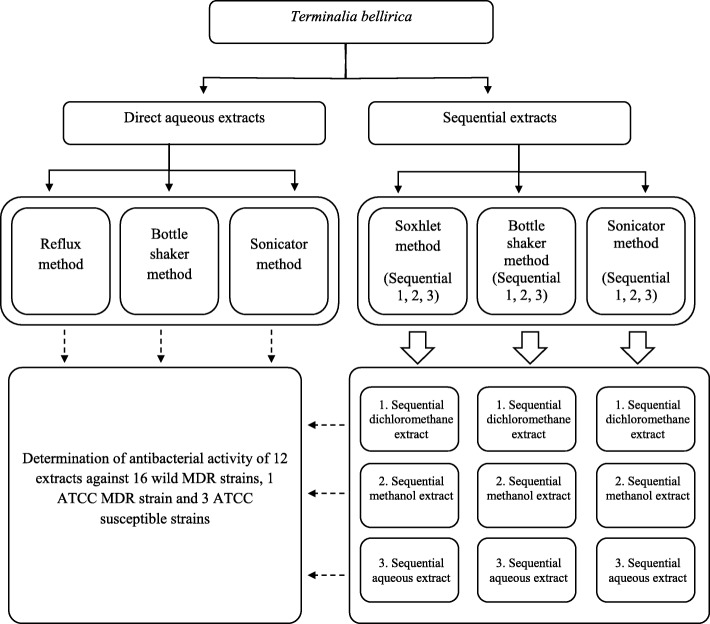
An overview of different methods used for extracting dried pericarp of *Terminalia bellirica* fruit

The fruits of *T. bellirica* were further dried in the laboratory at room temperature for 1 week and ground using an electric grinder to obtain powdered plant material. The plant extracts were prepared from the powdered plant material to obtain 3 direct aqueous extracts—using reflux, bottle shaker and sonicator methods—and 9 sequential extracts using Soxhlet, bottle shaker and sonicator methods (Fig. 1). Each sequential extraction procedure involved three solvents (dichloromethane, methanol and water) of increasing polarity. In each extraction procedure—direct aqueous or sequential—the ratio of dried fruit (weight in g) and extracting solvent (volume in mL) was maintained as 1:10 [26]. The procedures employed for the preparation of direct aqueous extracts and sequential extracts are given below.

#### Direct aqueous extracts

Aqueous extracts were obtained from the powdered plant material using the following three methods: an aliquot of the powdered material (40 g) was suspended in distilled water (400 mL) and heated under reflux for 6 h; a second aliquot of the powdered material (40 g) was also extracted into distilled water (400 mL) under ambient temperature using a bottle-shaker (GFL 3016, Germany) for 24 h; a third aqueous extract was obtained by ultrasound sonication (Branson 2510, USA) of the powdered material (40 g) in distilled water (400 mL) at ambient temperature for 3 h. Each aqueous extract was centrifuged, and the supernatant freeze-dried to obtain a light brown powder.

#### Sequential extracts

The powdered plant material (40 g) was packed in a cellulose thimble, placed in the extraction tube of a Soxhlet apparatus and extracted with dichloromethane (400 mL) for 6 h, and then the extraction was continued with methanol (400 mL) for a further 6 h. The plant residue was dried and heated in distilled water (400 mL) under reflux for 6 h. Another aliquot of powdered plant material (40 g) was also extracted into dichloromethane (400 mL) followed by methanol (400 mL) and distilled water (400 mL) at ambient temperature for 24 h using a bottle-shaker. A third set of sequential extracts was obtained by ultrasound sonication of the powdered material (40 g) in dichloromethane (400 mL) followed by methanol (400 mL) and distilled water (400 mL) at ambient temperature for 3 h using an ultrasound sonicator. The solvent of each dichloromethane and methanol extract was removed under reduced pressure using a rotary evaporator (Heidolph, Laborota 4000, Germany). Each sequentially obtained aqueous extract was centrifuged and the supernatant freeze-dried to obtain a crude powder. The powder obtained from each extract was checked for sterility on a nutrient agar plate and stored in air tight universal bottles at − 20 °C till further testing. A schematic diagram of the extraction procedures is given in Fig. 1.

### Screening for antibacterial activity

The aqueous and organic solvent extracts of *T. bellirica* fruits were screened for antibacterial activity using the cut-well diffusion method [26]. Briefly, bacterial suspensions of test and control organisms were adjusted to McFarland turbidity of 0.5 (approximately 1 × 10^8^ cfu/mL) and inoculated onto Mueller Hinton agar (MHA, Oxoid, Hampshire, England). The plates were left at room temperature for 30 min after which 12-mm diameter wells were bored in the agar and the bottom sealed with molten MHA. The organic extracts were dissolved with the aid of 10% (*v*/v) aqueous dimethyl sulfoxide (DMSO, BDH, England). Using a template, aliquots of each reconstituted extract (10 mg/mL) were pipetted into the wells and the plates incubated aerobically at 35 °C for 24 h. The diameter of the zone of inhibition (ZOI) around the well was measured along with the well. Each screening was carried out in triplicate and the mean diameter of the ZOI was recorded.

### Minimum inhibitory concentration (MIC)

The MICs of the aqueous and organic fruit extracts were determined by the agar dilution method [27]. Briefly, stock solutions of concentration 20 and 10 mg/mL were prepared from each extract and diluted with molten MHA (45 °C) to obtain a series of concentrations 5, 4, 2, 1, 0.5, 0.25 and 0.125 mg/mL, which were poured into sterile petri dishes and allowed to set. A 2-μL drop of each test and control organism prepared as stated above was inoculated onto each plate using a template. The plates were read after incubation at 35 °C for 24 h. The lowest concentration of extract that exhibited no visible growth was recorded as the MIC for each organism.

### Determination of DPPH free radical scavenging activity

DPPH radical (Sigma Aldrich, USA) scavenging activity was determined following a procedure described by Zhang et al. [28], with slight modifications. Briefly, 100 μL of each extract at various dilutions (50–3 ppm) was mixed with 100 μL of 1.6 mM DPPH solution in flat-bottom 96-well microtiter plates. The mixture was shaken for 1 min, kept for 30 min in the dark and the absorbance measured at 517 nm in an automated microplate reader (Biochrome UVM 340-Elisa Reader, USA). All determinations were performed in triplicate. L-ascorbic acid was used as a positive control.

The percentage scavenging effect was calculated as: % Scavenging rate = [{A_0_ − (A_1_ − A_2_)} / A_0_] × 100%, where A_0_ is the absorbance of the control (without sample) and A_1_ is the absorbance of sample in the presence of the DPPH, A_2_ is the absorbance of sample without DPPH radical (blank absorbance). The scavenging ability of the samples was expressed as EC_50_ value, the effective concentration at which 50% of DPPH radicals were scavenged; the EC_50_ value was calculated from the curve of percentage scavenging activity (%) versus concentration of the respective sample.

### Determination of total phenolic content (TPC)

TPC of solvent extracts of *T. bellirica* was determined using Folin-Ciocalteu reagent following a procedure described by Antolovich et al. [29], with minor modifications. Briefly, 20 μL of each extract was mixed with 100 μL of 1:10 Folin-Ciocalteu’s reagent (Merck, Germany) followed by the addition of aqueous Na_2_CO_3_ (80 μL, 7.5%). The assay was carried out in the automated microplate reader. After incubation at room temperature for 1 h in the dark, the absorbance at 765 nm was recorded. A standard curve for gallic acid solution (1, 2, 3, 4, 5, 6, 7, 8, 9, 10 and 20 ppm) was prepared using the same procedure. TPC was expressed as mg gallic acid equivalents per gram of dried extract (mg GAE/g).

### Cytotoxic assay of the most potent antibacterial extract of *T. bellirica*

Of the 12 extracts obtained from *T. bellirica* fruits, the direct aqueous extract (reflux method) was identified as the extract having the highest antibacterial potency. The cytotoxicity of this extract was evaluated using baby hamster kidney (BHK-21) cells available from the Animal Virus Laboratory, Veterinary Research Institute, Polgolla, Sri Lanka. The cytotoxic assay was performed by the method described by Jirasripongpun et al. [30], with minor modifications*.* Briefly, the BHK-21 cells (1 × 10^5^ cells/mL) were seeded onto a 6-well plate (Falcon, New Jersey, USA) containing Minimum Essential Medium Eagle (MEM) (Sigma-Aldrich, USA) supplemented with 10% fetal calf serum (Sigma Aldrich, USA) to provide confluence after 10–12 h incubation. The spent medium was removed, and the volume adjusted to 10 mL with new medium (MEM) containing a solution of the direct aqueous extract (reflux method) of *T. bellirica* such that the concentration of the extract was 4, 2, 1, 0.5, 0.25 and 0.125 mg/mL at separate runs. Distilled water and 20% aqueous DMSO (Sigma Aldrich, USA) containing plates served as negative and positive controls, respectively. The cultures were further incubated for 48 h and samples were counted for cell viability each day using Tryphan Blue exclusion method and hemocytometer. Each experiment was carried out in triplicate and averaged percent cell viability was plotted against concentration of *T. bellirica* aqueous extract. The 50% inhibition concentration (IC_50_) reflects the concentration of *T. bellirica* extract causing a 50% decrease in cell viability.

### Statistical analysis

All the experiments were carried out in triplicate. The data is expressed as mean ± standard deviation (SD). To determine the significant differences between values, analysis of variance (ANOVA) and Duncan’s multiple range tests were performed. Significance of difference was defined at 5% level (*p* < 0.05). All the statistical analysis was carried out using Excel (Microsoft Inc.), SigmaPlot10 (Systat Software, Inc., San Jose California, USA, http://systasoftware.com) and R-3.2.0 (R Software Inc. Vienna, Austria, http://www.r-project.org). The correlation analysis was performed between antioxidant activity (mean EC_50_) and total phenolic content.

## Results

The hot aqueous extraction of *T. bellirica* fruits by the reflux method followed by freeze-drying furnished a light brown powder with 21.4% yield (*w*/w). The corresponding yields of the aqueous extraction by the bottle-shaker and the ultrasound sonicator methods at ambient temperature were 16.8 and 13.2%, respectively. The yields of extracts obtained by Soxhlet method, bottle-shaker method and ultrasound sonication method in the sequential extractions using solvents of increasing polarity were respectively 0.4, 2.8 and 3% for dichloromethane extracts, 27.8, 17.6 and 14.6% for methanolic extracts, and 20.8, 5.5 and 4.3% for aqueous extracts.

### Antibacterial activity (cut-well diffusion method) of extracts

#### Direct aqueous extracts

**Fig. 2:**
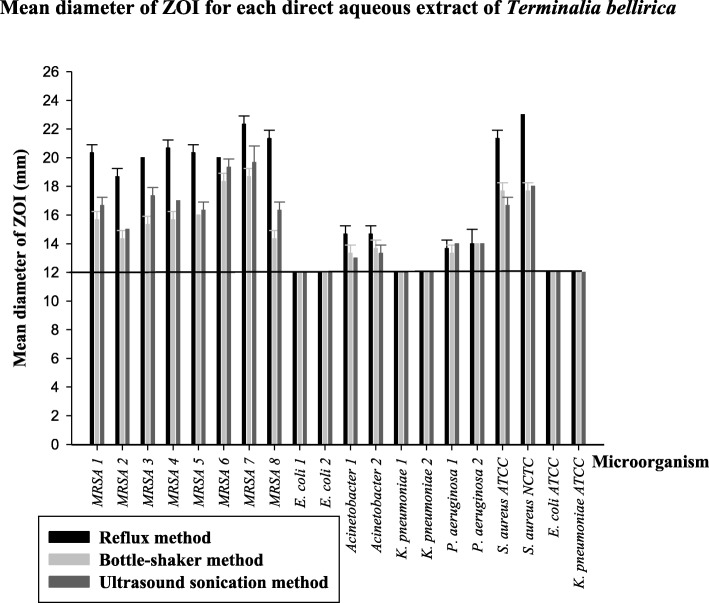
Mean diameter of zones of inhibition (ZOI) for direct aqueous extracts (10 mg/mL) of *Terminalia bellirica* prepared from reflux method, bottle-shaker method and ultrasound sonication method

The mean diameter of the zones of inhibition (ZOIs) with standard deviation for direct aqueous extracts (10 mg/mL) against the tested panel of bacteria (16 MDR wild strains and 1 ATCC MDR strain and 3 ATCC susceptible strains) is given in Fig. 2. The extracts obtained from the hot aqueous extraction procedure (reflux method) consistently displayed larger ZOIs against the eight MRSA strains (18–23 mm) and the two strains of MDR *Acinetobacter* spp. (14–15 mm) than the other two aqueous extracts obtained from bottle-shaker method and ultrasound sonication method while all three aqueous extracts displayed almost similar inhibition areas against the two strains of *P. aeruginosa* (13–14 mm).

#### Sequential dichloromethane extracts

None of the sequential dichloromethane extracts obtained by the three extraction methods showed an inhibition zone for any of the bacterial strains tested at 10 mg/mL.

#### Sequential methanol extracts

**Fig. 3:**
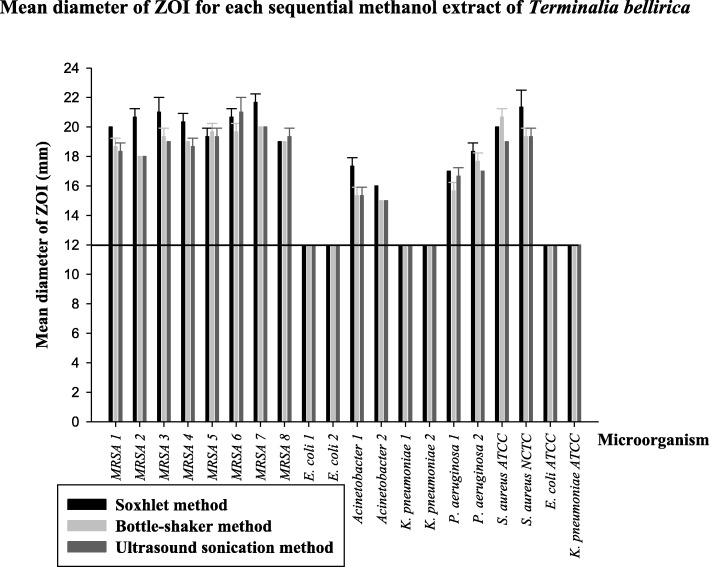
Mean diameter of zones of inhibition (ZOI) for sequential methanol extracts (10 mg/mL) of *Terminalia bellirica* prepared from Soxhlet method, bottle-shaker method and ultrasound sonication method

The screening results (mean diameter of ZOIs with standard deviation) for sequential methanol extracts (10 mg/mL) obtained from the three extraction methods are given in Fig. 3. All three extracts had comparable ZOIs although the sequential methanol extract from the Soxhlet method showed slightly larger inhibition zones against the eight MRSA strains (19–22 mm), the two strains of MDR *Acinetobacter* spp. (16–18 mm) and the two strains of MDR *P. aeruginosa* (17–19 mm) than those by the other two extracts.

#### Sequential aqueous extracts

**Fig. 4:**
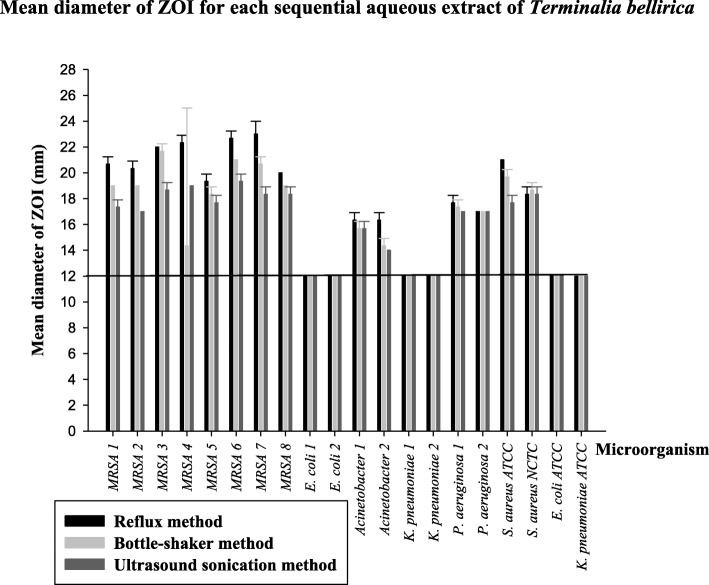
Mean diameter of zones of inhibition (ZOI) for sequential aqueous extracts (10 mg/mL) of *Terminalia bellirica* prepared from reflux method, bottle-shaker method and ultrasound sonication method

The screening results (mean diameter of ZOIs with standard deviation) for the sequential aqueous extracts (10 mg/mL) are given in Fig. 4. The sequential aqueous extract from Soxhlet method showed larger ZOIs than those from the other two extraction methods against the eight MRSA strains (20–23 mm) and the two MDR *Acinetobacter* spp. strains (16–17 mm). The three extracts showed similar ZOIs against the two MDR *P. aeruginosa* (16–18 mm) strains.

### MIC values

#### Direct aqueous extracts

The MIC values of the direct aqueous extracts of *T. bellirica* were determined for the same panel of bacteria by the agar dilution method and the results are given in Table 1. The direct aqueous extract obtained by the reflux method displayed comparatively lower MIC values than the two direct aqueous extracts prepared at ambient temperature (bottle-shaker and ultrasound sonication methods). The MIC value of the extract from the reflux method was 0.25 mg/mL for six MRSA strains, 0.5 mg/mL for the remaining two MRSA strains and 0.5 mg/mL for MDR *Acinetobacter* spp. and MDR *P. aeruginosa* strains. The MICs of the direct aqueous extracts against ESBL-producing *E. coli* and MDR *K. pneumoniae* strains originating from the clinical isolates were relatively high (> 5 mg/mL). The extracts did not produce a ZOI against these two species in the cut-well diffusion method as shown in Fig. 2.

**Table 1:** Minimum inhibitory concentration values (MIC) of *Terminalia bellirica* fruit extracts obtained by different extraction methods, against 16 MDR strains (from clinical isolates), 1 ATCC MDR strain and 3 ATCC susceptible strains

Microorganism^a^	Minimum inhibitory concentration (MIC) in mg/mL											
	Direct aqueous extracts			Sequential extracts								
	Reflux method	Bottle- shaker method	Sonication method	Soxhlet method			Bottle-shaker method			Sonication method		
				DCM^b^	Methanol	Aqueous	DCM	Methanol	Aqueous	DCM	Methanol	Aqueous
1	0.5	0.5	0.5	NA^c^	0.25	0.5	NA	0.5	0.5	NA	0.5	0.5
2	0.5	0.5	0.5	NA	0.25	0.5	NA	0.5	0.5	NA	0.5	0.5
3	0.25	0.5	0.25	NA	0.25	0.5	NA	0.5	0.5	NA	0.5	0.5
4	0.25	0.5	0.25	NA	0.25	0.5	NA	0.5	0.5	NA	0.5	0.5
5	0.25	0.5	0.25	NA	0.25	0.5	NA	0.5	0.5	NA	0.5	0.5
6	0.25	0.5	0.25	NA	0.25	0.5	NA	0.5	0.5	NA	0.5	0.5
7	0.25	0.5	0.25	NA	0.25	0.5	NA	0.5	0.5	NA	0.5	0.5
8	0.25	0.5	0.5	NA	0.25	0.5	NA	0.5	0.5	NA	0.5	0.5
9	> 5	> 5	> 5	NA	> 5	4	NA	> 5	4	NA	> 5	4
10	> 5	> 5	> 5	NA	> 5	4	NA	> 5	4	NA	> 5	4
11	0.5	1	1	NA	0.5	1	NA	1	1	NA	1	1
12	0.5	1	0.5	NA	0.5	1	NA	1	1	NA	1	1
13	> 5	> 5	> 5	NA	> 5	> 5	NA	> 5	> 5	NA	> 5	> 5
14	> 5	> 5	> 5	NA	> 5	> 5	NA	> 5	> 5	NA	> 5	> 5
15	0.5	4	1	NA	1	2	NA	4	2	NA	4	2
16	0.5	4	1	NA	0.5	1	NA	1	1	NA	1	1
17	0.25	0.25	0.5	NA	0.5	1	NA	1	1	NA	1	1
18	0.25	0.25	0.25	NA	0.5	1	NA	1	1	NA	1	1
19	> 5	> 5	> 5	NA	> 5	> 5	NA	> 5	> 5	NA	> 5	> 5
20	> 5	> 5	> 5	NA	> 5	> 5	NA	> 5	> 5	NA	> 5	> 5

^a^Microorganisms: 1–8 – MRSA; 9,10 - ESBL producing *E. coli*; 11,12 - MDR *Acinetobacter* spp.; 13,14 - MDR *K. pneumoniae*; 15,16 - MDR *P. aeruginosa*;17,18 - *S. aureus* ATCC 25923 and NCTC 6571; 19 - *E. coli* ATCC 25922; 20 - ESBL producing *K. pneumoniae* ATCC 700603

^b^DCM dichloromethane

^c^NA Not applicable

#### Sequential extracts

The sequential methanol extract from the Soxhlet method showed low MIC values: 0.25 mg/mL for MRSA strains, 0.5 mg/mL for MDR *Acinetobacter* spp. and 0.5–1 mg/mL for MDR *P. aeruginosa* strains (Table 1). The other two sequential methanol extracts and the three sequential aqueous extracts also demonstrated considerably high antibacterial activity against the same panel of MDR bacterial strains. None of the extracts inhibited the growth of MDR *K. pneumoniae* at or below a concentration of 5 mg/mL. The MIC value of the three sequential aqueous extracts for ESBL-producing *E. coli* strains was 4 mg/mL; however, the sequential methanol extracts did not inhibit the growth of the *E. coli* strains at or below a concentration of 5 mg/mL.

Summing up the results of antibacterial assays, all *T. bellirica* extracts, except the three sequential dichloromethane extracts, were active (Table 1) against the eight strains of MRSA (MIC, 0.25–0.5 mg/mL), the two strains of MDR *Acinetobacter* spp. (MIC, 0.5–1.0 mg/mL) and the two strains of MDR *P. aeruginosa* (0.5–4.0 mg/mL); MRSA strains showed the lowest MICs. Only the three sequential aqueous extracts were active against the two ESBL-producing *E. coli* (MIC, 4.0 mg/mL) and the other extracts did not show activity against these two strains at a concentration less than 5 mg/mL. The two strains of MDR *K. pneumoniae* were the most resistant to all the extracts (MIC > 5 mg/mL).

### DPPH free radical scavenging activity and TPC

**Fig. 5:**
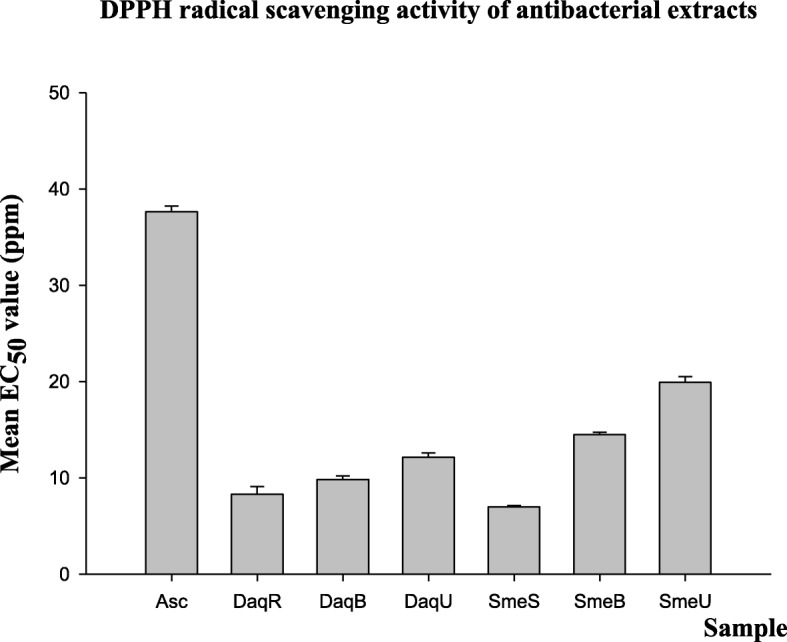
DPPH radical scavenging activity (EC_50_ value) of ascorbic acid and *Terminalia bellirica* extracts (Sample: Asc, Ascorbic acid; DaqR, direct aqueous extract from reflux method; DaqB, direct aqueous extract from bottle-shaker method; DaqU, direct aqueous extract from ultrasound sonication method; SmeS, sequential methanol extract from Soxhlet method; SmeB, sequential methanol extract from bottle-shaker method; SmeU, sequential methanol extract from ultrasound sonication method)

The antioxidant activity was determined for the three direct aqueous extracts and the three sequential methanol extracts obtained from Soxhlet, bottle-shaker and ultrasound sonication methods (Fig. 1). All the six extracts, having much lower EC_50_ values (Fig. 5), showed higher antioxidant activity than L-ascorbic acid.

The TPC was also determined for the above six extracts. The TPC values (Table 2), which were determined from the calibration curve prepared using gallic acid as the standard, varied from 63.36 ± 1.93 mg GAE g^− 1^ (sonicator methanol extract) to 188.71 ± 2.12 mg GAE g^− 1^ (Soxhlet methanol extract).

**Table 2:** Total phenolic content (TPC) of direct aqueous and sequential methanol extracts

Type of extract (Extraction method)	Extraction conditions	TPC (mg GAE/g)^a^
Direct aqueous (Reflux)	Hot, 6 h	135.52 ± 2.62b
Direct aqueous (Bottle)	Ambient, 24 h	118.80 ± 8.01b
Direct aqueous (Sonicator)	Ambient, 3 h	83.06 ± 4.56d
Sequential methanol (Soxhlet)	Hot, 6 h	188.71 ± 2.12f
Sequential methanol (Bottle)	Ambient, 24 h	95.26 ± 2.94c
Sequential methanol (Sonicator)	Ambient, 3 h	63.36 ± 1.93e

^a^Values are mean ± SD for three replicates; TPC is expressed as mg gallic acid equivalents per gram of dried extract

Mean values without letters (a, b, c, d and e) in common differ significantly (*p* < 0.05)

### Cytotoxicity to BHK-21 cells

The direct aqueous extract (reflux method) displayed the highest antibacterial activity, and this extract was assayed for cytotoxicity against BHK-21 cells using Tryphan Blue exclusion method [30]. The mean percent viablility of BHK-21 cells of three replicates for each concentration of extract corresponding to 24 and 48 h exposure is given in Table 3. A concentration-viability curve was drawn for each of the three replicates and IC_50_ values were calculated using the linear graph equations. IC_50_ values obtained for the three replicates were used to calculate the mean IC_50_ value with standard deviation. The mean IC_50_ values were 2.62 ± 0.06 and 1.45 ± 0.08 mg/ml for 24 and 48 h exposure, respectively.

**Table 3:** Cell viability of BHK-21 cells treated with direct aqueous extract (reflux) of *Terminalia bellirica* fruit

Concentration of extract (mg/mL)	% Cell viability^a^	
	After 24 h	After 48 h
4	0	0
2	71.20 ± 0.75	38.00 ± 0.20
1	85.30 ± 0.81	62.00 ± 0.87
0.5	93.20 ± 0.71	82.00 ± 1.21
0.25	100	87.50 ± 0.62
0.125	100	90.00 ± 1.77

^a^Values are mean ± SD for three replicates

## Discussion

In traditional medicine, decoctions based on *T. bellirica* are often prepared by heating the crushed fruit in boiling water. Under these conditions, thermolabile components, if present, may be denatured. To test this possibility, the extraction was performed under both hot and ambient conditions in the present study. To extract organic active principles that are not soluble in water, a sequential extraction protocol involving dichloromethane, methanol and water was introduced to the three different extraction methods (Fig. 1). The three extraction methods also represent hot (Soxhlet method) and ambient (bottle-shaker method and ultrasound sonication method) conditions. The ambient conditions were included to minimize thermal decomposition of thermolabile active principles, if any [26]. The 3 direct aqueous extracts and the 9 sequential extracts (dichloromethane, methanol and water) were subjected to antibacterial screening against 16 MDR bacterial strains that displayed resistance to 12 antibiotics selected from 6 different chemical classes belonging to penicillins, cephalosporins, carbapenems, quinolones, monobactams and aminoglycosides.

The observation that the sequential dichloromethane extracts were inactive against the tested panel of MDR bacteria even at a concentration of 10 mg/mL indicates that the active principles of *T. bellirica* are polar compounds that are not extractable to dichloromethane. The polar active principles are also thermostable because the methanol and aqueous extracts prepared under both ambient and hot conditions showed activity.

The Soxhlet procedure, compared to the other methods, offers exhaustive extraction of the plant material. The sequential methanol extract (Soxhlet) probably contained all the polar active principles that are soluble in hot methanol and consequently displayed higher antibacterial activity than the other two sequential methanol extracts (Table 1). The fact that the sequential aqueous extracts were also active reveals that *T. bellirica* fruit contains highly polar antibacterial compounds that are insoluble in methanol.

The direct aqueous extracts are expected to contain the water-soluble antibacterial compounds that would be present in both the sequential methanol and aqueous extracts. The water-solubility of the active compounds being enhanced under hot (reflux) conditions, the direct aqueous extract (reflux) showed high antibacterial activity compared to the other extracts.

Gram-negative bacteria to which *K. pneumoniae* and *E. coli* belong are usually less susceptible to antibiotics than Gram-positive bacteria because the outer membrane surrounding the cell wall in Gram-negative bacteria restricts diffusion of compounds through its lipopolysaccharide cover [31], and the periplasmic space in them contains enzymes that can break down foreign molecules introduced from outside [32]. Jinukti and Giri [33] report that an aqueous extract of *T. bellirica* fruits at 0.5 mg/mL inhibits drug-sensitive strains of both Gram-negative (*K. pneumoniae* MTCC 3384, *E. coli* MTCC 7410, *P. aeruginosa* MTCC 2295, *Proteus mirabilis* MTCC 425, *Salmonella typhimurium* MTCC 98 and *Proteus vulgaris* MTCC 744) and Gram-positive (*S. aureus* MTCC 7443 and *Bacillus sphaericus* MTCC 511) bacteria in an agar well-diffusion assay and that, among these microbial strains, *P. mirabilis* and *K. pneumoniae* have been the most sensitive. Thus, the relatively low susceptibility of MDR *K. pneumoniae* and MDR *E. coli* to *T. bellirica* aqueous extracts, compared to other organisms in the current study, may be attributed to the presence of multiple resistant mechanisms in the same isolate [4, 34] found in MDR lactose-fermenting Gram-negative bacteria. The non-lactose fermenters MDR *Acinetobacter* spp. and MDR *P. aeruginosa* were susceptible (Table 1) to *T. bellirica* aqueous extracts even though they, too, are Gram-negative bacilli.

The extracts that had antibacterial activity were also examined for antioxidant activity using the DPPH method; DPPH is a relatively stable free radical readily reducible by antioxidants. The DPPH method is routinely used as a valid, accurate and sensitive procedure to evaluate the radical scavenging activity of antioxidants [35]. All the extracts examined displayed significantly (*p* < 0.05) higher radical scavenging activity (EC_50_ 0.007–0.02 mg/mL) than L-ascorbic acid (EC_50_ 0.037 mg/mL); the value for L-ascorbic acid in the present study compared well with a value that has been reported in a previous study (0.043 mg/mL) [19]. The antioxidant activity appeared to vary with the method of extraction (Fig. 5). Interestingly, the antioxidant activity of the extracts obtained under hot conditions was higher (*p* < 0.05) than those obtained under ambient conditions, implying that the antioxidant principles, too, are thermally stable, and better extracted into hot solvents.

Along with the antioxidant activity, the TPC was also found to be higher in the extracts prepared under hot conditions than under ambient conditions (Table 2). The sequential methanol extract (Soxhlet) and the direct aqueous extract (reflux) that involved hot conditions recorded a TPC of 188.71 ± 2.12 and 135.52 ± 2.62 GAE mg/g, respectively, whereas the aqueous and sequential methanol extracts prepared under ambient conditions had values that ranged from 118.80 ± 8.01 to 63.36 ± 1.93 GAE mg/g depending on the extraction method and solvent. The TPC values in the present study compared well with those reported for methanol extracts in previous studies, 149.690 ± 6.088 GAE mg/g (methanol) [36], 111.67 ± 19.29 GAE mg/g (98% methanol) [37], 133 ± 0.003 GAE mg/g (70% methanol) [21] and 108.692 ± 7.914 GAE mg/g (50% methanol) [36].

**Fig. 6:**
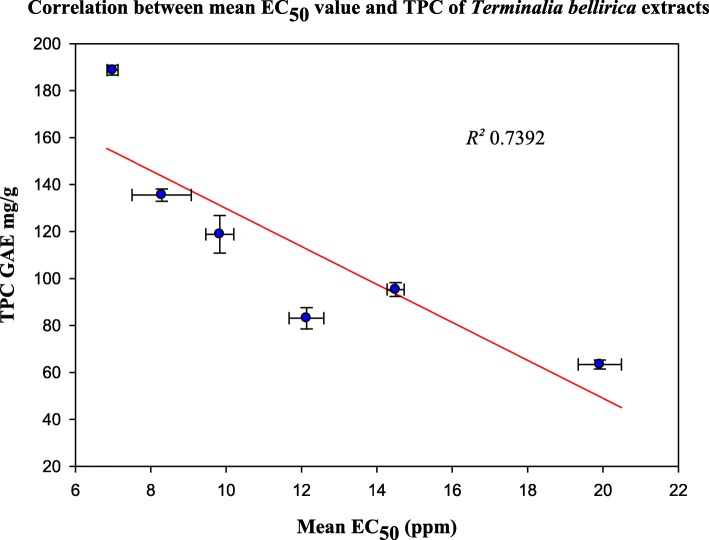
Relationship between total phenolic content (TPC in gallic acid equivalents, GAE) and EC_50_ values of DPPH scavenging activity

The TPC of the extracts correlated (*R*^*2*^ = 0.7392) with the corresponding antioxidant activity (Fig. 6), indicating that phenolic compounds are the major contributors to the antioxidant properties of *T. bellirica* fruit extracts [11, 19, 21]. Ethyl acetate soluble fraction of a methanol extract of *T. bellirica* fruit pericarp has shown high antioxidant and radical scavenging activity against DPPH, superoxide and hydroxyl radicals and the activity has been attributed to high phenolic and flavonoid content, gallic acid and ferulic acids being the major phenolics [20]. Correlations of antioxidant and radical scavenging activity with TPC have also been reported for extracts of other medicinal plants [37, 38].

There are a very limited number of studies on the evaluation of cytotoxicity of *T. bellirica* fruit. An aqueous extract prepared by boiling the fruit in water, has caused neither acute (at a single dose of 5000 mg/kg body weight) nor chronic (at a daily dose of 1200 mg/kg body weight over 270 days) toxicity in either female or male Spargue-Dawley rats [22]. The IC_50_ value of a hydroglycol extract of *T. bellirica* fruit against normal mouse fibroblast cells and mouse melanoma cells has been 5.43 mg/mL and 2.0 mg/mL at 48 h incubation, respectively [24]. We examined the aqueous extract, prepared by boiling the fruit in water under reflux, for cytotoxic effects on baby hamster kidney (BHK-21) fibroblast cells. A concentration response cytotoxicity curve with respect to time revealed a decrease of IC_50_ value from 2.62 ± 0.06 mg/mL at 24 h incubation to 1.45 ± 0.08 mg/mL at 48 h incubation. When determining toxicity of plant extracts using mammalian cell lines, a plant extract having an IC_50_ value ≥0.02 mg/mL is considered non-toxic or weakly toxic [39]. Thus, having large IC_50_ values on BHK-21 cells, the aqueous extract (reflux) of *T. bellirica* fruit, the most potent antibacterial extract, is unlikely to contain compounds lethal to normal mammalian cells.

## Conclusions

The methanol and aqueous extracts of the dried pericarp of *T. bellirica* fruits contained non-toxic polar antibacterial compounds with high thermal stability, which inhibited the growth of drug-resistant wild strains of *S. aureus*, *Acinetobacter* spp., *P. aeruginosa* and *E. coli*. The extraction of *T. bellirica* fruit in boiling water (heating under reflux) was found to be the most effective in terms of yield, antimicrobial potency and antioxidant potential of the extract. The antibacterial extracts possessed high antioxidant activity that correlated with the total phenolic content of the extracts. The most potent antibacterial extract against MDR organisms appeared non-toxic to baby hamster kidney (BHK-21) fibroblast cells and displayed high antioxidant activity exceeding that of L-ascorbic acid. Further studies on *T. bellirica* fruit extracts are warranted to develop effective, low cost, non-toxic antibiotics to combat MDR bacterial infections.

## Abbreviations

ATCC: American Type Culture Collection
BHK: baby hamster kidney
DMSO: dimethyl sulfoxide
EC_50_: 50% effective concentration
ESBL: extended spectrum β-lactamase
GAE: gallic acid equivalent
IC_50_: 50% inhibition concentration
MDR: multi-drug resistant
MEM: minimum essential medium
MHA: Mueller Hinton agar
MIC: minimum inhibitory concentration
MRSA: methicillin-resistant *Staphylococcus aureus*
MTCC: Microbial Type Culture Collection and Gene Bank
N/A: not applicable
NA: no activity
NCTC: National Collection of Type Cultures
SD: standard deviation
TPC: total phenolic content
*V*/V: volume to volume
WHO: World Health Organization
ZOI: zone of inhibition
